# Sugar from Rags

**Published:** 1881-09

**Authors:** 


### SUGAR FROM RAGS.

The newspapers have lately taken up the subject of making sugar from rags, and some of them seem to regard it as a new invention.

This, however, is by no means the case. It has been long known to chemists that if vegetable fiber, such as that of cotton, flax, etc., be submitted to the action of sulphuric acid, it is converted into soluble starch or dextrine, and this is readily convertible into sugar. The ordinary process of malting is simply a conversion of the starch of the barley into sugar by the agency of a ferment called "diastase," which is formed in the barley,and is so effective that only one five-hundredth part is sufficient to set up the action by which the insoluble starch is converted into dextrine, and then into sugar. This occurs when the grain of barley is sown in the ground, and is the natural operation by which the germ is fed : the germ having neither mouth nor stomach, cannot take solid food like the original starch granules which surround it in the seed ; but when that starch is converted into sugar, the baby plant can absorb it, and continues to absorb it until its rootlets and first leaf are formed. By this time the sugar is all used up, but the plant is now able to obtain its nourishment from the ground by its root, and from the carbonic acid of the air by its green leaf or leaves.

Such is the ordinary life history, not only of the barley plant, but of all others. The starch is to the plant germ what the yolk and white of the egg are to the chick germ. If the sugar were already formed in the seed it would be dissolved away at once by the water in the soil, and the germ would perish prematurely, but by the exquisite chemistry of nature the conversion of the insoluble starch into the soluble food of the germ goes on just so fast as the germ can use it, and thus the supply is kept up till the young plant can shift for itself. The malster forces the natural process, and then kills the germ by roasting the seed when he has obtained the maximum amount of sugar.

Fruits also are sugar factories, in which is conducted the whole process of making sugar from rags, the fiber of the rags being represented by the fiber of the unripe fruit. Every boy who has struggled to eat an unripe apple or pear knows that the unwholesome luxury is what he calls "woody," as well as sour. The chemist describes it similarly. His technical name for the tough material is "woody fiber," under which name he includes nearly all the fibrous materials of the vegetable world, for they all have fundamentally a similar chemical composition. This woody fiber is made up of carbon and the elements of water. Starch and sugar are composed of the same elements, their differences of properties being due to differences of arrangement and proportions of the constituent elements. Thus the change of insoluble starch into dextrine, and dextrine into sugar, or the change of woody fiber into dextrine and sugar, are effected by very small modifications of chemical composition.

We all know that the unripe apple or pear is sour, or that it contains an acid as well as the woody matter. Now, this appears to act after the manner of the sulphuric acid that the chemist applies to rags, but it acts more slowly and more effectively. The sweetest of pears are gathered when hard and quite unfit for eating, but by simply setting them aside and giving this acid time enough to do its work, the hard fibrous substance becomes converted into a delicious, sweet, juicy pulp.

The natural chemistry here has a great advantage over the artificial operation, seeing that the natural acid either becomes itself converted sugar, or combines with the basic substances in the fruit, forming wholesome salts. Not so the sulphuric acid of the chemist. He must get rid of this from his rag sugar; and herein lies the difficulty of the process. The writer tried the experiment more than twenty years ago, using lime for the purpose of removing the sulphuric acid, but found that in removing the sulphate of lime he lost much of the sugar which this solid absorbed, and from which it could only be removed by great dilution, and then not completely. To do this practically would cost so much that the rag sugar would be far dearer than that which nature beneficently manufactures by similarly, but more effectively, acting upon the fibers of the sugar cane or beet root.

There is little risk of the sugar trade being disturbed, or of the paper makers being deprived of their raw material, by the rivalry of rag sugar, though the chemist may display in a show glass some crystals that he has made from one of his own worn-out shirts.—*London Grocer.*

A Statute of Harvey has been completed by a London sculptor, and will be unveiled at Folkestone in August, during the session of the International Medical College.

